# Ochratoxin A in Dry-Cured Ham: OTA-Producing Fungi, Prevalence, Detection Methods, and Biocontrol Strategies—A Review

**DOI:** 10.3390/toxins14100693

**Published:** 2022-10-09

**Authors:** Yuanshan Chen, Jiang Chen, Qiujin Zhu, Jing Wan

**Affiliations:** 1 Department of Liquor and Food Engineering, Guizhou University, Huaxi District, Guiyang 550025, China; 2 College of Life Sciences, Guizhou University, Huaxi District, Guiyang 550025, China; 3 Key Laboratory of Plant Resource Conservation and Germplasm Innovation in Mountainous Region (Ministry of Education), College of Life Sciences/Institute of Agro-Bioengineering, Guizhou University, Huaxi District, Guiyang 550025, China

**Keywords:** dry-cured ham, Ochratoxin A, *Penicillium* spp., *Aspergillus* spp., detection methods, biological control

## Abstract

Traditional dry-cured hams are easily contaminated by toxigenic fungi during the fermentation and ripening stages. The detection and positive rates of ochratoxin A (OTA) are the highest among mycotoxins detected in traditional dry-cured hams, indicating that OTA in hams is a potential safety hazard to human health. This review addresses the mycotoxin-producing fungal species, the toxigenic conditions causing OTA contamination worldwide, the prevalence of OTA contamination in dry-cured hams, and the detection methods applied in OTA analysis. Additionally, this study introduces methods to prevent and control OTA in traditional dry-cured hams. The growth of common mycotoxin-producing fungi and the accumulation of mycotoxins in dry-cured ham can be controlled by a microbial starter. This review provides an important theoretical foundation for the research and control of OTA in traditional dry-cured hams.

## 1. Introduction

Dry-cured ham has a long manufacturing history and is one of the most popular traditional foods in various regions of the world, particularly those located in the Mediterranean region. Italy is the main producer of dry-cured hams, with annual sales of up to 30 million, of which San Daniele ham is famous for its quality and its typical “guitar”-like shape [1]. Spain has the second largest pig industry in the world, with a high consumption of Iberian pig meat. Dry-cured hams from Iberian black pigs display premium quality characteristics, with an average consumption of 2.04 kg per person per year in 2017 [2]. In China, Jinhua dry-cured ham and Panxian dry-cured ham represent the well-known traditional Chinese dry-cured meat products with natural fermentation periods. These hams are processed from the hind legs of local pigs over several months to years. The characteristic flavor of the dry-cured ham develops during this long ripening process [3].

Though dry-cured hams are popular with consumers, there is also a growing concern about their quality and safety. The preparation of traditional dry-cured hams made of fresh or frozen pork meat generally consists of four basic stages: salting, drying, natural fermentation, and ripening. The fermentation and ripening stages play indispensable roles in the formation of the unique flavor and quality of dry-cured ham. During the fermentation and ripening stages, various microorganisms colonize the surface of the hams as a suitable microbial culture medium under proper environmental conditions. Depending on the dominant species, some microorganisms benefit the flavor or antioxidant and protective functions, whereas others cause detrimental effects, such as undesirable odors/flavors, spoilage, and mycotoxin contamination.

Mycotoxins are secondary metabolites produced by toxigenic fungi under favorable conditions, which are acutely or chronically toxic to human and animals. About 500 mycotoxins have been identified [4]. Most mycotoxins contaminate plant-based food materials, such as cereals, grapes, coffee, nuts, and fodder. It is generally accepted that animal raw materials are clean of mycotoxin contamination, as mycotoxins are rigorously controlled for in fodder, so that mycotoxins entering meat in the food chain are blocked. However, due to the long natural fermentation stage, the fermentation conditions and physicochemical properties of fermented meat products are suitable for the growth of fungi, including toxigenic fungi, which results in a wide array of mycotoxins in traditional dry-cured meat products and fermented sausages. Therefore, dry-cured hams and fermented sausages provide a new exposure pathway for mycotoxin hazards other than plant-based foods. The common mycotoxins found in meat products are ochratoxin A (OTA), aflatoxins (AFs), deoxynivalenol (DON), zearalenone (ZEA), patulin (PAT), and citrinin (CIT). Among them, OTA is the most common, with a much higher detection level in traditional dry-cured hams, and is catching the attention of consumers and researchers. OTA has the structure of a phenylalanyl derivative of a substituted isocoumarin. Due to the thermodynamic stability of OTA during general food processing, it is very difficult to remove OTA once it has contaminated the food. OTA is the most toxic compound in the ochratoxin family. Because of the biological effects, including nephrotoxicity, hepatotoxicity, neurovirulence, immunotoxicity, carcinogenicity, teratogenicity, and mutagenicity [5], OTA contamination in dry-cured meat must be well controlled. In addition, OTA has been rated as a Group 2B carcinogen by the International Agency for Research on Cancer [6]. The Joint FAO/WHO Expert Committee on Food Additives announced that the provisionally tolerable weekly intake of OTA is 112 ng/kg BW/week [7], and it is a mycotoxin that has attracted worldwide attention after aflatoxin B1.

OTA was first identified from *Aspergillus ochraceus* in South Africa, from which it derived its name. Studies on OTA in dry-cured ham started in the 1970s. Escher et al. [8] isolated *Aspergillus ochraceus* and *Penicillium viridicatum* for the first time from country hams in Georgia and verified their OTA biosynthetic ability in in vitro media. However, scientists at that time only focused on the negative effects of mycotoxins on animal health through contaminated feed mildew, and ignored that the processing of meat products may also be a path for mycotoxins that cause damage to humans. In 1996, Gareis [9] systematically studied the composition and potential toxicity of surface mold during the ripening process of Spanish Iberian hams, and showed a high biological risk from surface molds on these hams. This result put the risk of mycotoxins from dry-cured hams into the public spotlight once again. Subsequent studies focused on identifying OTA-producing strains and confirmed their suitable growing and metabolic conditions [10]. In contrast, other researchers analyzed the OTA-producing ability of fungal species isolated from Istrian ham, and reported that there was no risk in the ham because no OTA-producing capacity was found in the isolated strains [11]. The establishment of an OTA detection method in 2002 for dry-cured ham using immunoaffinity clean-up combined with a quick fluorometric measurement promoted the popularization of precisely detecting OTA in meat and meat-derived products [12]. Subsequently, OTA was detected more frequently in ham and other cured meat products, which once again attracted the attention of scientists. However, the nature of the mycotoxins in dry-cured ham has always been controversial. Some have suggested that the presence of mycotoxins in dry-cured ham can be traced back to exposure to contaminated feed during the animal feeding period. Others believe that mycotoxins are synthesized by toxigenic fungi colonizing the hams during the fermentation and ripening periods. Some studies have indicated that the concentration of OTA in the raw materials was lower [13], whereas it was higher in dry-cured products [14], from which it was speculated that ham was being contaminated with OTA-producing fungi during the fermentation and ripening process. In 2010, Dall’Asta et al. [15] systematically studied the origin of OTA in dry-cured meat products. In an experiment, pigs were fed a sub-chronic level of OTA at the feeding stage for 40 days and processed into hams. The results showed that the OTA content in the hams was much higher than that in the raw meat. This was the first time it was demonstrated that the OTA in dry-cured ham was mainly caused by the colonization of toxigenic fungi on the surface of ham during the ripening stage, whereas the risk of OTA migrating from strictly controlled feed to ham was negligible.

**Figure 1:**
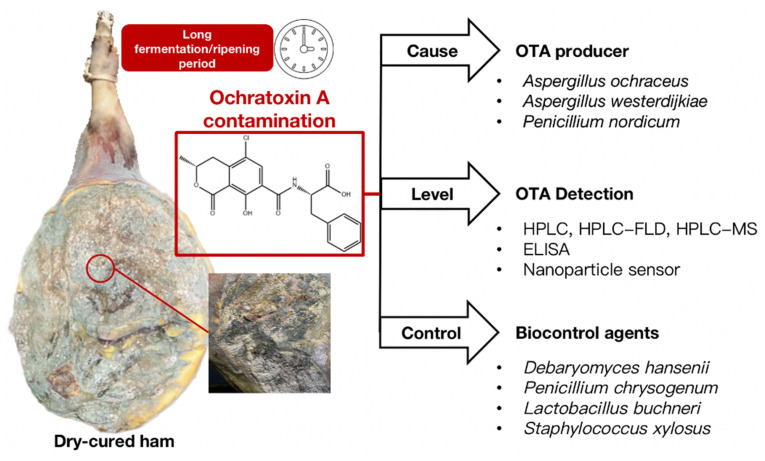
Outline of OTA-producing fungi, main detection methods, and biological control strategies in dry-cured ham.

It is now well accepted that OTA is a major food safety issue prevalent in the dry-cured meat industry worldwide. In addition, dry-cured ham produced by traditional families or workshops is more seriously contaminated with OTA than that produced by industry. For example, the highest OTA concentration detected in Iberian ham in Spain was 160.9 μg/kg [16]. In Italy, the Ministry of Health recommended that the maximum permissible level of OTA in meat or meat products would be 1 μg/kg in 1999 [17]. However, there is no legislation in the USA, Asia, Africa, or any other European country indicating the maximum amount of OTA allowed in meat products [18,19]. This review mainly focuses on: (i) the OTA-producing fungi in dry-cured ham and the suitable conditions needed for OTA synthesis, (ii) the prevalence of OTA contamination in dry-cured hams, (iii) the detection methods applied to analyze OTA, and (iv) the potential strategies to biocontrol OTA-producing fungal growth and OTA production in dry-cured ham (Figure 1).

## 2. OTA-Producing Fungi in Dry-Cured Hams

*Aspergillus* and *Penicillium* grow and reproduce on the surface of traditional dry-cured hams. Many species from these genera produce OTA and theoretically cause a potential OTA safety hazard in hams. The OTA-producing fungi that have been detected in dry-cured hams are *A. ochraceus*, *A. niger*, *A. steynii*, *A. subramanianii*, *A. westerdijkiae*, *P. brevicompactum*, *P. chrysogenum*, *P. commune*, *P. cyclopium*, *P. nordicum*, *P. polonicum*, and *P. verrucosum*. Because fungi possess the prominent features of the region, climate, and the colonizing matrix, new OTA producers are constantly being isolated from dry-cured hams worldwide [20,21].

OTA is mainly produced by *Aspergillus* spp. and *Penicillium* spp. Seven *Aspergillus* species have been identified to produce OTA: *A. ochraceus*, *A. alliaceus*, *A. melleus*, *A. auricomus*, *A. ostianus*, *A. petrakii*, and *A. sclerotioru* [22]. Subsequently, Frisvad et al. [23] reported that other species can produce OTA: *A. westerdijkiae*, *A. cretensis*, *A. flocculosus*, *A. roseoglobulosus*, *A. pseudoelegans*, and *A. sulphurous*. Among OTA-producing *Penicillium* spp., *P. verrucosum* and *P. nordicum* colonize the surface of dry-cured hams during the ripening stage, and were the first OTA-producing fungal species to be identified [24,25]. Then, *P. commune* and *P. chrysogenum* were determined to be OTA producers [26,27]. Table 1 displays the species and toxigenic conditions of the OTA producers and their growth and mycotoxin-producing conditions on dry-cured hams from all regions of the world. The most common fungi among them are *P. nordicum*, *P. verrucosum*, *A. ochraceus*, and *A. westerdijkiae*.

Fungi reproduce in large numbers during the ripening and fermentation processes of dry-cured hams. Therefore, the relationship between environmental factors (temperature and water) and the biosynthesis of mycotoxins has been studied extensively. The OTA-producing Aspergillus spp. and Penicillium spp. easily infect the surface of hams by synthesizing OTA at a suitable temperature and water activity (aw) [30].

The OTA-producing *Aspergillus* strains in dry-cured hams include *A. ochraceus*, *A. westerdijkiae*, *A. niger*, and *A. steynii*. Escher et al. [8] reported that three *A. ochraceus* strains isolated from traditional dry-cured hams produce OTA up to 7900 μg/kg at a temperature of 25 °C, humidity of 45%, and NaCl concentration of 4.1%. Rodriguez et al. [29] studied the influence of humidity on the OTA-producing ability of *A. ochraceus*, and suggested that *A. ochraceus* produces more OTA under lower humidity conditions (84% vs. 97%). In addition, a study by Wang et al. [31] indicated that the ochratoxigenic ability of *A. ochraceus* was effectively inhibited when the concentrations of NaCl and glucose were >40 and 150 g/L, respectively, in the culture medium. The effect of aw and temperature on fungal growth and OTA production by *A. westerdijkiae* isolated from dry-cured ham have been studied using a dry-cured ham-based medium. *A. westerdijkiae* grows over a wide range of conditions, but the growth rate of *A. westerdijkiae* accelerates as the temperature increases. The optimal OTA-producing conditions for *A. westerdijkiae* are 0.94–0.97 aw and 20–25 °C. The concentration of OTA detected under these conditions was 1943 μg/kg. No OTA was detected at 0.85 aw [21].

Dry-cured hams are naturally fermented and ripened over 12 months under controlled conditions, which promotes the colonization of fungi on the surface of the hams. The surface of dry-cured hams fermented at a relatively low temperature is often colonized by *Penicillium* spp. [7].

The OTA-producing *Penicillium* stains in dry-cured hams include *P. verrucosum*, *P. nordicum*, *P. nalviogense*, *P. commun*, *P. chrysogenum*, and *P. polonicum* [26,32]. Among them, *P. verrucosum* and *P. nordicum* are the most common species detected in dry-cured ham. The high level of NaCl in some traditional dry-cured hams poses a penetrative threat to influence the adaptability of the specific fungi on the surface of dry-cured hams [33,34]. Many studies have investigated the growth and OTA-producing characteristics of *P. verrucosum* and *P. nordicum* on the surface of dry-cured ham [16,21,35,36]. Rodríguez et al. [34] showed that the growth rate of *P. verrucosum* was faster than that of *P. nordicum* (*p* ≤ 0.05) under all test conditions. The best conditions for the growth of *P. verrucosum* were 0.94 aw and 25 °C, whereas they were less than 0.94 aw and 20 °C for *P. nordicum*. These results indicate that *P. nordicum* prefers to grow at a lower temperature than *P. verrucosum* [30]; that is to say, it is a natural adaptation for dry-cured hams to ferment and ripen at a lower temperature. Andrade et al. [37] inoculated *P. nordicum* on sliced dry-cured hams with 0.84 aw for fermentation. After 15 days of fermentation, the OTA level of the sliced ham was 42.93 μg/kg. In addition, different strains from the same fungal genus can produce significantly different quantities of OTA. For example, the OTA levels produced by two strains of *P. nordicum* were 15.99 μg/kg by *P. nordicum* strain 15 and less than the LOD by *P. nordicum* strain 110.769 in a dry-cured ham-based medium without NaCl or exogenous OTA [18]. This result was attributed to an intraspecific difference between the strains. Vipotnik et al. [21] reported that *P. nordicum* exhibits its lowest growth rate at 30 °C, and its growth rate increases with decreasing temperature. Moreover, temperature and water work together to affect the OTA-producing ability of fungi. About 712 μg/kg OTA was produced by *P. nordicum* in a dry-cured ham-based medium at 20 °C and 0.97 aw.

## 3. The Prevalence of OTA Contamination in Dry-Cured Ham

Dry-cured meat products, such as dry-cured hams, are traditional foods produced and consumed worldwide, which require strict quality controls to ensure consumer safety. OTA is the most frequently detected mycotoxin in dry-cured ham, and is related to the occurrence of several human diseases. Many studies have reported that the development of Balkan endemic nephropathy is associated with OTA [38].

In the past, dry-cured hams were produced in households where the production process was carried out under uncontrolled conditions, which led to questions about the safety and quality of the final product. Subsequently, standard industrial production was gradually developed to improve the production environment for dry-cured ham, systematize production, and increase the safety of the product. However, Table 2 shows that OTA has been detected in households and industrially produced dry-cured hams, and even up to 100% in manufactured hams. In addition, Dall’Asta et al. [15] and Vulić et al. [39] reported that dry-cured hams from the market contain OTA. As the origin of these dry-cured hams was unknown, either households or industrial production can contaminate dry-cured hams with OTA. In conclusion, whether the dry-cured hams are from households or industrial production, specific molds colonize the surface of the ham and produce OTA as long as the fermentation temperature and humidity are appropriate.

In addition to concerns about OTA residues on the surface of dry-cured ham, the spreading ability of OTA should be considered. The ripening environment and rich nutrition of dry-cured hams promote the surface colonization of specific mold populations, but mycotoxins can penetrate the meat. Rodríguez et al. [16] investigated the accumulation of OTA in Iberian dry-cured hams during mold growth and found that some deep parts of the ham were also infested with OTA. Similarly, Toscani et al. [40] and Dall’Asta et al. [15] detected the presence of OTA in the interior and external layers of hams, suggesting a migration pattern for OTA in dry-cured hams. The OTA level was always higher in the external layer than in the interior layer, which could be directly related to the OTA-producing fungi growing on the surface of the hams.

High OTA contamination rates have been frequently reported in dry-cured ham. Table 2 summarizes the detection method, the positive incidence rates, and the levels of OTA in dry-cured hams. About 17 to 100% of the dry-cured hams are contaminated with OTA worldwide.

It can be inferred that the varying concentrations of OTA in dry-cured hams from different countries can be linked to production technology, the climate of the producing region, or the detection method. Thus, controls must be implemented to minimize the risk of mycotoxin infection due to the consumption of these products.

## 4. OTA Detection Methods for Dry-Cured Hams

The OTA detection technology and quantitative methods for dry-cured hams are constantly upgraded and reported. The common methods include enzyme-linked immunosorbent assay (ELISA), high-performance liquid chromatography (HPLC), and high-performance liquid chromatography-mass spectrometry (HPLC-MS), as shown in Table 2.

The ELISA method fixes an antigen or antibody on the surface of a carrier to maintain activity. An enzyme-labeled antigen or antibody is added to specifically bind with the antigen or antibody. The specific binding of the antigen to the antibody results in the high sensitivity and specificity of the ELISA, which is suitable for detecting a large sample set. For example, Zadravec et al. [30] examined the OTA levels in Croatian dry-cured hams (*n* = 92) using an ELISA with a limit of quantification (LOQ) of 1.72 μg/kg and a limit of detection (LOD) of 0.91 μg/kg. The results showed that 18.47% of the hams were OTA-positive, and the highest OTA level was 6.86 μg/kg. Additionally, Pleadin et al. [43] investigated the occurrence of OTA in different traditional meat products (*n* = 410) circulating in Croatian markets using ELISA. The LOQ and LOD for hams (*n* = 105) were 1.56 and 0.85 μg/kg, respectively. Among the dry-cured hams, the maximum observed OTA level (9.95 μg/kg) was about 10 times higher than the maximum recommended level for pork products set in some EU countries. However, an ELISA using zymoprotein may have a high false-positive rate, which must be carefully eliminated through a complicated pretreatment.

HPLC was developed in the late 1960s and is a common method to monitor various mycotoxins in food and feed materials. The working principle of HPLC is based on the adsorption ability of different samples in the liquid and solid phases, resulting in different retention times and peak areas for qualitative and quantitative analyses. HPLC plays an important role in mycotoxin analysis due to its high sensitivity, repeatability, and high precision. Bertuzzi et al. [42] established an HPLC method for detecting OTA with a LOD value of 0.10 μg/kg and a LOQ value of 0.04 μg/kg. They reported that the OTA concentration was >100 μg/kg on the surface of dry-cured ham. Chiavaro et al. [12] tested hams (*n* = 42) with fermentation periods of 6 and 12 months using an HPLC method with an average recovery of 74.6%, and 15 samples had OTA levels > 1 μg/kg. However, HPLC can produce extra-column effects. In addition, this method is complex and expensive due to the elaborate sample preparation and standards needed.

The MS method is increasingly being used to detect mycotoxins in food. LC-MS can draw the gas chromatography-electron impact mass spectrometry by collecting mass spectrometry, but it could not provide more fragment ions of the compound and detailed structural information of the molecule. LC-MS can be used for quantitative, but not qualitative, analyses. LC-MS/MS is currently the most common way to detect mycotoxins. After samples flow through the sampling system, they enter the chromatographic column and are separated. Then, the sample enters the mass spectrum detector. LC-MS/MS produces molecular and fragmented ion peaks, and can be used for quantitative and qualitative analyses. This method has the features of high sensitivity and selectivity, a low detection limit, and high accuracy, so it can be used to monitor a variety of mycotoxins simultaneously. Rodriguez et al. [16] adopted HPLC-MS to analyze OTA in Iberian dry-cured hams. As results, the LOD was 1 μg/kg, the LOQ was 3 μg/kg, and the OTA recovery was > 90%. Peromingo et al. [46] used LC-MS/MS to detect several secondary mycotoxin metabolites produced during the dry-cured ham ripening stage. The detection and qualitative limits were 0.29 and 1.0 μg/kg, respectively. LC-MS has greater accuracy and higher sensitivity than other methods, but the cost of detection is higher.

Due to the disadvantages of these assays, such as the high cost of instrumentation, the complex sample preparation, the time-consuming processes, and the susceptibility to matrix interference [47,48], emerging rapid immunoassays, including fluorescence polarization immunoassay, chemiluminescence immunoassay, surface plasmon resonance immunosensing, electrochemical immunosensing, surface-enhanced Raman scattering immunosensing, and colorimetric aptamer sensors, have been proposed [49,50,51,52,53,54]. Although these immunoassay technologies show excellent performance and efficiency in detecting mycotoxins, several issues, such as poor sensitivity and bio-selectivity, high quantitative requirements, and the observation that some methods do not achieve the purpose of point-of-need rapidly, are big hurdles to commercializing such sensing techniques [48,55].

Moreover, the use of electrochemical sensors and biosensors results in great attention due to the facilities, such as ease of operation, short assay time, and high sensitivity. However, this practical application has remained in the laboratory, and actual samples are examined by a standard addition technique rather than batch recognition of OTA [56]. A versatile Y-shaped DNA nanostructure has been developed for simple, rapid detection of OTA. The Y-shaped duplex DNA arms were formed with two DNA tweezers at the ends. The aptamer sequence at the third end binds to the target mycotoxin with strong affinity and releases the two DNA fragments. The amount of OTA can be quantitatively detected by recovering the fluorescent intensity. This method has been utilized to detect OTA in food samples with satisfactory results, thus exhibiting a promising future in food safety screening and quality monitoring [57]. Altunbas et al. [58] presented the first example of a Tb3+-functionalized nanoparticle sensor for rapid, sensitive, and selective fluorescent detection of OTA. A microparticle-counting immunosensor has been recently developed based on polydopamine nanoparticle-mediated click chemistry to detect OTA by Chen et al. [59]. This method has high sensitivity and accuracy; however, the assay is incapable of detecting multiple targets simultaneously.

In general, it remains a challenge to exploit faster, lower-cost, and stable methods for detecting mycotoxins.

## 5. Biocontrol Strategies

The OTA detected in dry-cured hams has potential health consequences for consumers; thus, it is crucial to develop strategies to remove OTA from the food chain. However, OTA is very stable in dry-cured ham, making it difficult to eliminate. Physical measures, such as heating and drying, do not effectively reduce OTA content in ham. Instead, these physical measures may change the flavor and texture quality of the dry-cured ham [60]. In addition, chemical methods cannot be used to prevent OTA due to the undesired chemical residues and instability of the dry-cured ham products. Although some chemical germicides inhibit OTA-producing fungi, they can also suppress the growth of beneficial microorganisms on the surface of the hams. Furthermore, consumers worry about chemical residues in food [61]. Therefore, physical and chemical measures are unsuitable to control OTA in dry-cured hams.

Therefore, biocontrol methods are the most promising measures to prevent OTA production and accumulation by creating competition among the microorganisms. Many studies have shown that OTA contamination can be effectively prevented by using fine strains screened from dry-cured hams as biocontrol agents. This biocontrol method is considered safe, healthy, and effective. OTA concentrations have been reduced or inhibited in dry-cured ham and other meat products by using biological control agents, as shown in Table 3. Yeast, a common natural microbial flora on the surface of traditional dry-cured hams, contributes to the unique flavor of ham, and is a biocontrol candidate to restrain the growth of OTA producers [61]. *Debaryomyces hansenii* is the most important yeast during the production of traditional dry-cured hams, which belongs to the list of Qualified Presumption of Safety (QPS) by the European Food Safety Authority [62]. *D. hansenii* is utilized as a starter culture to enhance the safety of dry-cured hams by competing for nutrition and space with OTA producers, producing extracellular compounds, and influencing secondary metabolism [63]. Peromingo et al. [64] reported that the OTA content in dry-cured hams decreases by 80% when the hams are inoculated with yeast strains. Simoncini et al. [65] screened yeast strains from dry-cured hams, and monitored their ability to inhibit the growth and OTA biosynthesis of *P. nordicum.* The results showed that *D. hansenii* strain 147 had the strongest inhibitory activity against *P. nordicum*.

The non-toxigenic molds on dry-cured hams are often the source of biocontrol strains against OTA accumulation on dry-cured hams. In some traditional dry-cured hams, *Penicillium* spp. are the dominant microbes on the surface of hams during the ripening stage, which include beneficial *Penicillium* spp., as well as toxigenic *Penicillium* spp. [66]. One *P. chrysogenum* strain (RP42C) significantly reduces OTA contamination in dry-cured hams by secreting the antifungal protein, PgAFP [66,67]. *P. chrysogenum* strain RP42C has also been applied during the ham-curing process. The results showed that the OTA contamination rate of dry-cured hams treated with *P. chrysogenum* strain RP42C decreased from 33% to 0% [36]. Furthermore, when only *P. nordicum* was inoculated on a dry-cured ham-based medium, the OTA content was 5 ng/g, which decreased five times after inoculating with *P. chrysogenum* [68]. Another study showed that the antifungal effect was strengthened if both *P. chrysogenum* and *D. hansenii* were used. The average OTA concentration in the control group was 1620 μg/kg, whereas the average OTA concentration in the group inoculated with *P. chrysogenum* and *D. hansenii* was 11.73 μg/kg, and the incidence rates of positive hams decreased from 75% to 33% [69]. These results show the potential for the mixed protective cultures to effectively control OTA contamination in dry-cured hams. *D. hansenii* and *P. chrysogenum* can also shorten ripening time, improve flavor and color, and stabilize the quality of dry-cured ham.

A few studies have focused on a biocontrol agent screened from Gram-positive, catalase-positive cocci. Cabezón et al. [70] isolated *Staphylococcus xylosus* Sx8 from dry-cured hams, and verified its antifungal ability. After OTA-producing fungi *P. nordicum* 15 or *P. nordicum* 92 were inoculated with *S. xylosus* at different temperatures, the OTA content produced by *P. nordicum* was less than the LOD and the LOQ. However, the OTA content of the sample co-inoculated with both *P. nordicum* 856 and *S. xylosus* increased at a particular temperature. Therefore, the environmental conditions and the specific toxigenic strains should be considered, although utilizing *S. xylosus* to inhibit OTA producers and OTA concentrations in dry-cured hams is a promising biological control method.

Lactobacillus (LAB) are microorganisms produced during food fermentation that synthesize a variety of antifungal compounds and prevent the growth of a broad range of fungi. LAB can be used as a protective culture to improve the microbiological safety of food products without changing their sensory characteristics. LAB have GRAS (Generally Regarded as Safe) and QPS status, given by the US Food and Drug Administration and the European Food Safety Authority, respectively [71]. The biopreservation ability of different LAB strains on coffee, fruits, and vegetables has been demonstrated [72,73,74]. Only Iacumin et al. [75] reported *Lactobacillus buchneri* and *D. hansenii* as potential biological preservatives that could be used to inhibit OTA production by *A. westerdijkiae* in dry-cured hams. Nevertheless, there is a lack of studies on antifungal LAB against OTA in dry-cured hams. This approach provides researchers with new ideas to prevent or reduce the growth of ochratoxigenic molds and the production of OTA. Notably, in most in vivo biocontrol studies, the ham was first sterilized and then inoculated with OTA-producing fungi, as well as a biocontrol strain, to observe the effect of the biocontrol strain on the growth and OTA accumulation of OTA-producing fungi. However, interestingly, the growth and OTA accumulation of wild-type ochratoxigenic molds were controlled in a study by Rodríguez et al. [36] by applying the biocontrol strains directly to the surface of naturally contaminated dry-cured ham.

A comprehensive understanding of the mode of action of antagonistic microorganisms is useful to improve their performance against toxigenic molds, and to establish screening criteria for more effective strains. The major mechanisms of action of bacteria, yeasts, and non-toxigenic molds isolated from dry-cured hams include the production of volatile antifungal compounds and competition for nutrients and space [36,37,70]. In addition, yeasts may also reduce mycotoxin levels by adsorbing cell wall molecules, such as glycomannoproteins [76], or by blocking the biosynthetic pathway of mycotoxins [77].

**Table 3:** Biocontrol strains used to reduce ochratoxin A in dry-cured meat products.

Biocontrol Strains	OTA Producer	Food Product	Inhibition Rate (%)	Reference
*D. hansenii*	*P. nordicum*	Dry-cured pork	96.27	[65]
*D. hansenii*	*P. nordicum*	Dry-cured ham slice	65	[37]
*Saccharomycopsis fibuligera*	*A. ochraceus*	Speck	100	[78]
*D. hansenii*	*A. ochraceus*	Speck	100	[78]
*D. hansenii*	*P. verrucosum*	Dry-cured ham	80	[64]
*P. chrysogenum* and *D. hansenii*	*P. nordicum*	Dry-cured ham	98.51	[69]
*D. hansenii*	*P. nordicum*	Dry-cured ham	68.24	[68]
*P. chrysogenum*	*P. nordicum*	Dry-cured ham	80	[68]
*D. hansenii* and *L.buchneri*	*A. westerdijkiae*	Dry-cured ham	100	[75]
*S. xylosus*	*P. nordicum*	Dry-cured ham-based media	86.59	[70]
*D. hansenii*	*P. nordicum*	Cured meat	100	[63]
*D. hansenii*	*P. nordicum*	Dry-cured fermented sausage	54.97	[79]

Furthermore, consideration should also be given to the effect of biocontrol agents on ham quality, such as the flavor characteristics. However, limited information is available. To our knowledge, only short-seasoned dry-cured hams have been evaluated for the effect of *D. hansenii* and *L. buchneri* as biological agents on the sensorial acceptability of ham. The results showed no sensorial differences between the biocontrol treatment group and the control group [75]. Iacumin et al. [78] explored the inhibitory activity of yeast on OTA production during speck production, and performed a sensory analysis. The results also showed no differences between batches, and excellent taste in all groups. An evaluation of the side effects of biocontrol agents on the quality profile of ham will play an essential role in the final application.

## 6. Conclusions

OTA contamination of dry-cured hams is very common, and poses potential food safety hazards. The main OTA producers isolated and identified from dry-cured hams are *P. verrucosum*, *P. nordicum*, *A. ochraceus*, and *A. westerdijkiae*. The diversity of OTA producers in dry-cured hams can be affected by many factors, such as the manufacturing technique and the environmental conditions, so the diversity has significant regional, climatic, and colonization matrix specificity. Therefore, new OTA-producing fungi are constantly being isolated and identified from dry-cured hams worldwide. Currently, the study of OTA biosynthesis, preventive strategies, and limit standards are rare worldwide and mainly depend on research from European and American countries. Dry-cured ham is a type of traditional natural fermented food with a long history of production that needs to be studied from more diverse regions according to a specific geography, climate, and processing mode. Due to the uniqueness of the raw materials and ingredients, as well as the manufacturing technique, of traditional dry-cured hams, screening safe biocontrol agents is one of the most promising measures to control OTA in traditional dry-cured ham. Among the microorganisms usually present on dry-cured ham, yeasts, such as *D. hansenii*, play an important role in the fermentation of dry-cured ham, particularly during the drying–ripening stage, by providing certain sensory characteristics to the product. It is feasible to screen additional microorganisms for biocontrol, verify their effectiveness in vitro and in vivo, and apply them to the product. However, in addition to the ability of biocontrol agents to inhibit OTA, the effect of certain species on the flavor quality of dry-cured ham during fermentation should also be considered.

## Figures and Tables

**Table 1:** OTA producer and their growth and mycotoxin-producing conditions in dry-cured ham.

Hams Species	OTA Producer	Growing Condition		Toxigenic Condition		Reference
		Temperature (°C)	Water Activity (Aw)	Temperature (°C)	Water Activity (Aw)	
American country ham	*A.ochraceus*	25–30	/	25–30	/	[8]
Italy dry-cured ham	*P. nordicum*	25	0.95–0.98	25	0.98	[28]
Spanish dry-cured ham	*P. nordicum*	25	0.84	25	0.92	[29]
	*A.ochraceus*	25	0.84	25	0.92	
Portugal dry-cured ham	*A. westerdijkiae*	25–30	0.93–0.97	20–25	0.94–0.97	[21]
	*P. nordicum*	19–25	0.93–0.97	18–22	0.95–0.97	

/: the OTA-producing fungi were isolated and identified, but there was a lack of the growth and OTA biosynthesis characteristics of the fungal strain.

**Table 2:** OTA contamination of dry-cured ham.

Country	Source	Sample Volume	Incidence of Positives (%)	Mean of Positives (μg/kg)	Concentration Range (μg/kg)	LOD (μg/kg)	Analytical Method	Reference
Italy	manufacturer	21	80.95	1.22	0.20–2.20	0.04	HPLC	[12]
	manufacturer	30	40	1.62	0.01–28.42	0.01	HPLC-FLD	[14]
	market	5	60	2.67	<0.02–7.28	0.02	HPLC-FLD	[40]
	market	110	76.36	0.53	0.10–12.51	0.1	HPLC-FLD	[15]
	manufacturer	40	100	3.27	1.14–6.29	0.06	HPLC	[41]
	household	6	83.33	21.40	<0.04–104	0.05	HPLC	[42]
Spanish	manufacturer	20	50	27.1	2.00–160.90	1	HPLC-MS	[16]
	household	15	33.33	3.20	2.00–6.30	1	HPLC-MS	[36]
Croatia	household and market	105	17.14	0.77	0.97–9.95	0.15	ELISA/HPLC-FLD	[43]
	market	54	22.22	3.16	1.56–9.95	0.32	ELISA	[39]
	household	67	22.38	4.3	2.16–6.86	0.91	ELISA	[30]
Bosnia	household	8	25	3.29	2.11–4.47	1.5	ELISA	[44]
Portugal	manufacturer	47	42.55	14.90	<0.90–99.10	0.3	HPLC-FLD	[45]

HPLC: high-performance liquid chromatography; HPLC-FLD: high-performance liquid chromatography-fluorescence detector; HPLC-MS: high-performance liquid chromatography-mass spectrometry; ELISA: enzyme-linked immunosorbent assay.

## Data Availability

Not applicable.
